# The Probiotic Properties and Safety of *Limosilactobacillus mucosae* NK41 and *Bifidobacterium longum* NK46

**DOI:** 10.3390/microorganisms12040776

**Published:** 2024-04-11

**Authors:** Jaekoo Lee, Jaehyun Jo, Hanseul Seo, Seung-Won Han, Dong-Hyun Kim

**Affiliations:** 1 PB Business Department, NVP Healthcare Inc., Suwon 16209, Republic of Korea; jklee@nvp-healthcare.com (J.L.); jhjo@nvp-healthcare.com (J.J.); hsseo@nvp-healthcare.com (H.S.); swhan@nvp-healthcare.com (S.-W.H.); 2 Department of Food Regulatory Science, Korea University, Sejong 30019, Republic of Korea; 3 Neurobiota Research Center, College of Pharmacy, Kyung Hee University, Seoul 02447, Republic of Korea

**Keywords:** probiotics, *Limosilactobacillus mucosae*, *Bifidobacterium longum*, probiotic properties, safety

## Abstract

Probiotics should possess specific properties to exert beneficial effects, and their safety must be ensured for human consumption. The purpose of this study was to evaluate the probiotic properties and safety of *Limosilactobacillus mucosae* NK41 and *Bifidobacterium longum* NK46 isolated from human feces in vitro. Both strains exhibited high resistance to simulated gastrointestinal fluid. Furthermore, probiotic-related cell surface characteristics including auto-aggregation and cell surface hydrophobicity were assessed by measuring the absorbance at a wavelength of 600 nm, which demonstrated good auto-aggregation ability and affinity for xylene, indicating their effective adhesion to Caco-2 cells. In addition, hemolytic, gelatinase, and β-glucuronidase activities were found to be negative in both strains. The susceptibility to nine commonly used antibiotics was assessed using the broth macrodilution method, which demonstrated that both strains were susceptible to all tested antibiotics. Furthermore, *L. mucosae* NK41 and *B. longum* NK46 produced significantly higher levels of L-lactate (71.8 ± 0.7% and 97.8 ± 0.4%) than D-lactate (28.2 ± 0.7% and 2.2 ± 0.4%, respectively). Using PCR amplification to investigate genes associated with virulence factors, we found that neither strain harbored any virulence genes. These findings suggest that *L. mucosae* NK41 and *B. longum* NK46 have the potential to be used as probiotics and are considered safe for human consumption.

## 1. Introduction

Probiotics are live microorganisms that exert health-promoting effects on the host when consumed in moderate amounts [1]. In recent clinical trials, probiotics have been reported to have various beneficial effects, such as enhancement of immune function [2], alleviation of allergic rhinitis [3], improvement of postprandial lipid levels [4], amelioration of menopausal symptoms [5], maintenance of vaginal health [6], and anti-obesity [7]. Owing to their health benefits, the use of probiotics in food and pharmaceutical preparations has attracted considerable attention. The global probiotic market, including probiotic foods, probiotic drinks, and dietary supplements, has experienced strong growth owing to rising consumer awareness of the role of healthy functional foods in the gut microbial ecosystem [8].

The World Health Organization (WHO) has established guidelines for the evaluation of probiotics in food, including criteria for their efficacy and safety assessment [9]. Additionally, a series of in vitro tests have been devised and employed as criteria for selecting potential probiotics [8]. Among these, a few prerequisites for selecting probiotics for human consumption include resistance to gastrointestinal stress, adherence to gut epithelial cells, and safety aspects, such as the absence of hemolysis, antimicrobial sensitivity, and non-production of virulence factors and biogenic amines [10,11].

Potential probiotic strains should have certain characteristics, such as survival and adhesion abilities, under the gut environmental conditions for gastrointestinal colonization [12]. Probiotic isolates should be able to withstand the low pH of the stomach and survive under highly acidic conditions. Similarly, probiotic strains require bile tolerance to successfully pass through the small intestine. Additionally, the ability of probiotic strains to adhere to the intestinal epithelial cells is crucial for their colonization and persistence in the gastrointestinal tract (GIT) [11]. The adhesion mechanisms of probiotics involve complex processes, including nonspecific physical binding such as hydrophobicity and interactions with specific cell wall components [13]. Therefore, prior to using probiotic strains, their survival in the GIT and adherence to the epithelium should be confirmed by in vitro evaluation.

Many species of lactic acid bacteria (LAB) are generally recognized as safe (GRAS) by the Food and Drug Administration (FDA) because of their long history of safe consumption [14]. In addition, most species belonging to the genera formerly known as *Lactobacillus* and *Bifidobacterium* meet the qualified presumption of safety status established by the European Food Safety Authority (EFSA) and are commercially used [15,16]. However, these safety properties are strain-specific and cannot be generalized to all LAB strains. LAB, particularly *Enterococcus* spp., can transfer virulence and antibiotic-resistant genes to other bacteria via horizontal gene transfer. Considering these characteristics, every regulatory authority requires safety verification at the genetic level for approval of commercial applications of novel probiotic strains [17,18]. Therefore, the safety of new probiotic strains must be ensured, and risk factors must be adequately evaluated for human consumption [19].

*Limosilactobacillus mucosae* (*L. mucosae*) NK41 and *Bifidobacterium longum* (*B. longum*) NK46, which were selected from a human fecal bacterial collection, have been previously reported to induce the expression of brain-derived neurotrophic factor, which promotes the proliferation, survival, and differentiation of neurons in corticosterone-stimulated SH-SY5Y cells [20,21]. Moreover, these strains improve cognitive impairment-like behavior and neuroinflammation in aged or 5xFAD transgenic mice [21], and reduce anxiety and depression and colitis by suppressing gut dysbiosis in immobilization stress-induced mice [20]. The aim of this study was to assess the probiotic properties (resistance to gastrointestinal conditions, auto-aggregation, hydrophobicity, adhesion to intestinal epithelial cells, and extracellular enzyme activity) and safety characteristics (hemolysis, gelatinase activity, antimicrobial susceptibility, production of D-/L-lactate, and identification of genes associated with virulence factors) of these strains for potential commercial applications as functional foods.

## 2. Materials and Methods

### 2.1. Bacterial Strains and Cultivation Conditions

*L. mucosae* NK41 and *B. longum* NK46 strains were obtained from the Korea Culture Center of Microorganisms (Seoul, Republic of Korea). These strains were inoculated into De Man, Rogosa, and Sharpe (MRS) broth (Difco Laboratories, Detroit, MI, USA) and incubated at 37 °C under anaerobic conditions. *Staphylococcus aureus* (ATCC 25923) and *Enterococcus faecalis* (ATCC 29212 and ATCC 19433), which serve as quality control strains, were provided by the Korean Collection for Type Cultures (KCTC, Daejeon, Republic of Korea) and were used as positive controls in the safety studies. The pathogenic strains were grown in brain heart infusion broth (Difco Laboratories) at 37 °C under aerobic conditions.

### 2.2. Cell Culture

Caco-2 cells were obtained from the Korea Cell Line Bank (Seoul, Republic of Korea). Caco-2 cells were maintained in Dulbecco’s modified Eagle’s medium (DMEM; Gibco, Grand Island, NY, USA) supplemented with 10% heat-inactivated fetal bovine serum (FBS; Gibco) and 1% antibiotic-antimycotic solution (Gibco). The cell cultures were incubated at 37 °C in a humidified atmosphere containing 5% CO_2_. The cells were sub-cultured when they reached 80% confluence.

### 2.3. Resistance to Simulated Gastrointestinal Environments

The resistance of the probiotic strains to a continuous model of simulated gastrointestinal fluid was investigated according to the method described by Lee et al. [22], with slight modifications. The simulated gastric fluid (SGF) was simulated in phosphate-buffered saline (PBS; Gendepot Inc., Baker, TX, USA) with 0.3% pepsin (Sigma, St. Louis, MO, USA), and the pH was adjusted to 2.5 using 1 M HCl (Daejung Chemical Co., Ltd., Shiheung, Republic of Korea). To prepare simulated intestinal fluid (SIF), 1% (*w*/*v*) pancreatin (Wako, Osaka, Japan) and 1% (*w*/*v*) bile salt (Difco Laboratories) were added to the PBS and adjusted to pH 7.4 using 0.2 M NaOH (Daejung Chemical Co., Ltd.). One milliliter of bacterial suspension containing approximately 1 × 10^9^ CFU/mL was mixed with 9 mL of SGF and incubated at 37 °C for 2 h. Following a two-hour exposure to SGF, the cell pellets were collected by centrifugation (4000× *g* for 20 min), mixed with 10 mL of stimulated intestinal fluid, and followed by incubation at 37 °C for an additional 2.5 h. Bacterial viability was determined by the plate counting method and survival rate was calculated using the following Equation (1):

Survival rate (%) = (Log N/Log N_0_) × 100 (1)

where Log N represents the logarithm of the number of viable cells after exposure to the test conditions, and Log N_0_ represents the logarithm of the number of initial viable cells before exposure to the test conditions.

### 2.4. Auto-Aggregation

Auto-aggregation assays for the probiotic strains were conducted according to the method described by Rastogi et al. [23], with some modifications. Briefly, the probiotic strains were inoculated into MRS broth and incubated at 37 °C for 18 h under anaerobic conditions. After centrifugation at 4000× *g* for 20 min, the bacterial culture-derived cell pellets were washed twice with PBS and resuspended in the same buffer to adjust the absorbance at 600 nm to 1.0. The suspension was vortexed and incubated at 37 °C for 5 h. Every hour, the absorbance of the upper phase of the suspension was measured at 600 nm. The auto-aggregation percentage was calculated using the following Equation (2):

Auto-aggregation (%) = [1 − A_t_/A_0_] × 100, (2)

where A_t_ is the absorbance at various time points (1, 2, 3, 4, and 5 h) and A_0_ is the absorbance at 0 h.

### 2.5. Cell Surface Hydrophobicity

To assess bacterial cell surface hydrophobicity, a bacterial adhesion to the hydrocarbon assay was performed according to the method reported by Rastogi et al. [23]. Briefly, probiotic strain cultures were centrifuged at 4000× *g* for 20 min, washed twice with PBS, and resuspended in the same buffer to adjust the absorbance at 600 nm to 1.0. A total of 3 mL of the cell suspension was mixed with 1 mL of hydrocarbon xylene (Duksan Pure Chemical Co., Ltd., Ansan, Republic of Korea), and the mixture was thoroughly vortexed for 2 min. After incubating at 37 °C for 1 h, the optical density of the aqueous phase was measured at 600 nm. Cell surface hydrophobicity (%) was calculated using the following Equation (3):

Cell surface hydrophobicity (%) = [(A − B)/A] × 100,(3)

where A and B are the absorbance values before and after the organic solvent extraction, respectively.

### 2.6. Cell Cytotoxicity Assay

The cytotoxicity of the tested probiotic strains was determined using a lactate dehydrogenase (LDH) cytotoxicity assay kit (Biomax, Seoul, Republic of Korea) following the manufacturer’s protocols. Briefly, Caco-2 cells were seeded into 96-well plates (1 × 10^4^ cells/well). Subsequently, the probiotic strains were treated at different concentrations (10^7^ or 10^9^ CFU/mL) for 24 h. Cell lysis buffer was used as a positive control. Subsequently, 50 μL of supernatants obtained by centrifugation were mixed with 50 μL of substrate mix, and the reaction was carried out for 30 min in the dark. Absorbance at 490 nm was measured using an Epoch 2 microplate spectrophotometer (Bio-Tek, Winooski, VT, USA), and cytotoxicity was calculated using the following Equation (4):

Cytotoxicity (%) = [(A − B)/(C − B)] × 100,(4)

where A represents the absorbance of the supernatant from Caco-2 cells treated with the probiotic strains, B represents the absorbance of the supernatant from untreated cells, and C represents the absorbance of the supernatant from the positive control.

### 2.7. Adhesion to Caco-2 Cell Line

The ability of the probiotic strains used in this study to adhere to the intestinal epithelial cell line Caco-2 was determined following the method reported by Fonseca et al. [24], with slight modifications. Caco-2 cells precultured in DMEM supplemented with FBS and antibiotic-antimycotics were plated in 12-well plates (1 × 10^5^ cells/mL) and maintained at 37 °C for 24 h. For the adhesion assay, the probiotic strains were cultured in MRS broth at 37 °C for 18 h and then centrifuged (4000× *g* for 20 min). After washing the cell pellets twice with PBS, they (concentration: 1 × 10^8^ CFU/mL) were resuspended in DMEM without FBS and antibiotic-antimycotics. Bacterial cell suspension was added to each well, followed by incubation at 37 °C for 2 h in a humidified atmosphere containing 5% CO_2_. Subsequently, the cells were washed thrice with PBS to remove non-adherent bacteria and lysed with PBS containing 0.1% (*v*/*v*) Triton X-100 (Daejung Chemical Co., Ltd.). The number of bacteria attached to the cells was quantified by the plate counting method. The adhesion ability was represented as the percentage ratio between the initially added bacteria and the number of adherent bacterial cells.

### 2.8. Enzymatic Activity Profile

The enzymatic activities of probiotic strains were determined using the API ZYM test kit (bioMérieux, Marcy-l’Étoile, France) according to the manufacturer’s protocols. Briefly, bacterial cells were suspended in PBS to a concentration of 5.0–6.0 McFarland standard. The cell suspension (65 μL) was added to each cupule and incubated at 37 °C for 4 h. Subsequently, one drop of ZYM A and ZYM B reagents was added to each cupule. After 5 min, the results were evaluated using an established standard response chart provided by the manufacturer.

### 2.9. Hemolytic Activity

The hemolytic activity of the probiotic strains used in this study was assessed on blood agar (Kisan Biotech, Seoul, Republic of Korea) supplemented with 5% sheep blood. Overnight cultures of probiotic strains were inoculated on blood agar plates and the plates were incubated at 37 °C for 48 h under anaerobic conditions. Hemolytic activity was assessed based on the halo zones formed around the colonies. The presence of a greenish, clear, or no halo surrounding the colonies was respectively considered as α-hemolysis, β-hemolysis, or γ-hemolysis. *S*. *aureus* ATCC 25923 was used as a positive control for β-hemolysis.

### 2.10. Gelatinase Activity

The gelatinase activity of probiotic strains used in this study was determined on agar plates containing gelatin (Duksan Pure Chemical Co., Ltd.) according to the method reported by Veljović et al. [25] with some modifications. A drop of overnight cultures of each probiotic strain was deposited on MRS agar supplemented with 3% (*w*/*v*) gelatin, followed by incubation at 37 °C for 48 h. After incubation, the plates were flooded with 550 g/L ammonium sulfate solution. The presence of a transparent halo around the colonies was considered indicative of gelatinase production. *S*. *aureus* ATCC 25923 was used as a positive control and was incubated on tryptic soy agar (Difco Laboratories) containing 3% (*w*/*v*) gelatin.

### 2.11. Determination of Minimum Inhibitory Concentration

The minimum inhibitory concentrations (MICs) of the antibiotics against the probiotic strains were determined using the macrodilution broth method [26]. Nine antibiotics of human and veterinary importance, namely, ampicillin, vancomycin, gentamicin, kanamycin, streptomycin, erythromycin, clindamycin, tetracycline, and chloramphenicol (Sigma) were tested. Briefly, bacterial suspensions were inoculated at a concentration of 5 × 10^5^ CFU/mL in the LAB susceptibility test medium (Isosensitest broth: MRS broth = 9:1) supplemented with a series of antibiotic concentrations. Subsequently, the mixture was incubated at 37 °C for 24 h. The MICs were determined as the lowest concentration at which no visible growth was observed. According to the guidelines of the European Committee on Antimicrobial Susceptibility Testing and the Clinical and Laboratory Standards Institute (CLSI), limitations exist on the cut-off values for *Lactobacillus* and *Bifidobacterium*. Therefore, the results were interpreted based on the established cut-off values provided by the EFSA [27].

### 2.12. Determination of D(−)-Lactate and L(+)-Lactate

The amounts of D(−)-lactate and L(+)-lactate produced by the probiotic strains were determined using a D-/L-lactate assay kit (Abcam, Cambridge, UK) according to the manufacturer’s protocol. Briefly, 50 μL of supernatants separated from bacterial cultures were mixed with 46 μL of assay buffer, 2 μL of substrate mix, and 2 μL of enzyme mix. The reaction mixture was then incubated at room temperature for 30 min. Subsequently, the absorbance was measured at 450 nm using an Epoch 2 microplate spectrophotometer.

### 2.13. Detection of Genes for Virulence Factors Production

To verify the presence of genes encoding virulence factors, PCR amplification was performed using previously reported primers and conditions. The primers used to amplify these genes are listed in Table 1. Genomic DNA was extracted from the bacterial isolates using a DNA extraction kit (Bioneer, Daejeon, Republic of Korea) according to the manufacturer’s instructions. Genomic DNA was amplified under the following conditions: 94 °C for 5 min; followed by 30 cycles of 94 °C for 1 min, 52 °C (*gelE*, *efaA*), 56 °C (*esp*), and 48 °C (*ace*) for 30 s, and 72 °C for 1 min; and a final extension at 72 °C for 5 min. PCR products were separated by electrophoresis on 1.5% (*w*/*v*) agarose gels in 0.5× Tris-acetate-EDTA buffer (Bioneer). *E*. *faecalis* ATCC 29212 and ATCC 19433 were used as quality control strains.

### 2.14. Statistical Analysis

All data are presented as the mean ± standard deviation from three independent experiments. Statistical analyses were conducted using the SPSS software version 21 (IBM Inc., Armonk, NY, USA). The significance of the differences between the means was assessed using a two-tailed Student’s *t*-test following an analysis of variance.

## 3. Results and Discussion

### 3.1. Stability of the Probiotic Strains under Simulated Gastrointestinal Conditions

The strains that are to be used as probiotics must survive the harsh conditions of the GIT to exert health-promoting effects on the host [28]. A series of in vitro assessments such as resistance to acids, bile salts, and pancreatin have been regarded as reliable indicators for evaluating the probiotic properties of bacterial isolates [11]. In this study, the GIT resistance of *L. mucosae* NK41 and *B. longum* NK46 was investigated under a continuous model of simulated gastrointestinal fluid (Table 2). After exposure to simulated gastrointestinal conditions for 4.5 h, the survival rate of *L. mucosae* NK41 (71.5 ± 0.8%) was significantly higher than that of *B. longum* NK46 (40.8 ± 1.4%). In particular, both strains showed a decrease in cell viability after exposure to SGF, ranging from 1.7 to 4.6 Log CFU/mL. In contrast, cell viability hardly decreased after exposure to SIF containing 1% (*w*/*v*) pancreatin and 1% (*w*/*v*) bile salts. Consistent with these results, previous studies have reported that the survival rates of some strains of *L. mucosae* and *B. longum* significantly decreased after exposure to SGF but showed minimal reduction after exposure to SIF [29,30]. Several studies have reported that commercial probiotic strains, including *Lactobacillus* spp. and *Bifidobacterium* spp., have excellent survival rates under gastrointestinal conditions [31,32]. However, in these studies, the bacteria were incubated in a culture medium or peptone water to enhance their survival. Although the present study did not include substances that protect the strains in the simulated gastrointestinal fluid, both strains exhibited a survival rate of >40%, which is similar to the results of previous studies [28]. These results suggest that *L. mucosae* NK41 and *B. longum* NK46 may survive and persist in the GIT.

### 3.2. Auto-Aggregation and Cell Surface Hydrophobicity of the Probiotic Strains

**Figure 1:**
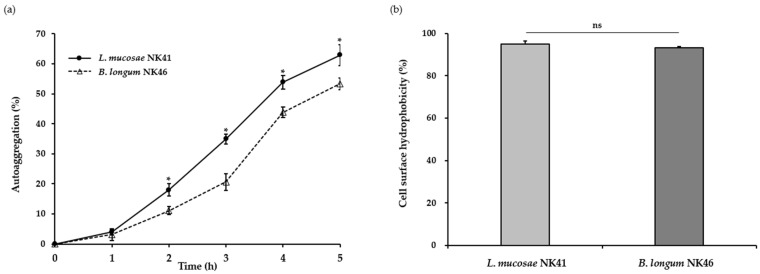
Auto-aggregation (**a**) and cell surface hydrophobicity (**b**) of *L. mucosae* NK41 and *B. longum* NK46 under simulated gastrointestinal conditions. Asterisks (*) denote significant differences from the means (*p* < 0.05) analyzed using Student’s *t*-test. ns: not significant.

Generally, auto-aggregation is considered a desirable property for probiotic functions. This enables probiotic bacteria to form aggregates and increase their adhesion to the intestinal epithelium [33]. Moreover, this characteristic provides protection against pathogenic bacteria by suppressing their adherence to the surface of the intestinal mucosa [34]. In this study, the aggregation abilities of *L. mucosae* NK41 and *B. longum* NK46 were assessed by spectrophotometric assays. As shown in Figure 1a, the auto-aggregation of both strains steadily increased over time. After incubation at 37 °C for 5 h, the auto-aggregation values of *L. mucosae* NK41 and *B. longum* NK46 were 62.9 ± 3.6% and 53.3 ± 1.9%, respectively. In a previous study, Tuo et al. [35] reported that the auto-aggregation of *Lacticaseibacillus rhamnosus* GG, a commercial probiotic strain, was 41.4 ± 3.3%. Furthermore, some *Lactobacillus* spp. and *Bifidobacterium* spp. exhibited low auto-aggregation abilities of 11.5–29.0% after 6 h of incubation [36].

The interaction between bacterial and epithelial cells is significantly influenced by cell surface hydrophobicity [36]. Proteins, teichoic acid, and lipoteichoic acid on the bacterial cell wall impart hydrophobic properties to the bacterial cell surface, whereas polysaccharides render the bacterial surface hydrophilic. In this regard, cell surface hydrophobicity contributes to cell adherence properties [34]. In this study, we conducted the bacterial adhesion to the hydrocarbon assay to assess the cell surface hydrophobicity of *L. mucosae* NK41 and *B. longum* NK46, and the results are presented in Figure 1b. Both *L. mucosae* NK41 and *B. longum* NK46 exhibited hydrophobicity values of 95.0 ± 1.6% and 93.2 ± 0.8%, respectively. According to previous studies by Rastogi et al. [23] and Purkayastha et al. [37], *L. mucosae* SRV5, SRV10, and K76 exhibit hydrophobicity ranging from 46.8 to 57.7%. In addition, Krausova et al. [36] reported that *B. longum* SL5B had a low value of hydrophobicity of 39.2 ± 13.5%. In a previous study, auto-aggregation and cell surface hydrophobicity were strongly correlated with the adhesion ability to Caco-2 cells [38]. The strains tested in the present study exhibited higher auto-aggregation ability and hydrophobicity compared to the results of previous studies. Therefore, these findings suggest that *L. mucosae* NK41 and *B. longum* NK46 have the potential to be used as commercial probiotics.

### 3.3. Adhesion Ability of the Probiotic Strains

**Figure 2:**
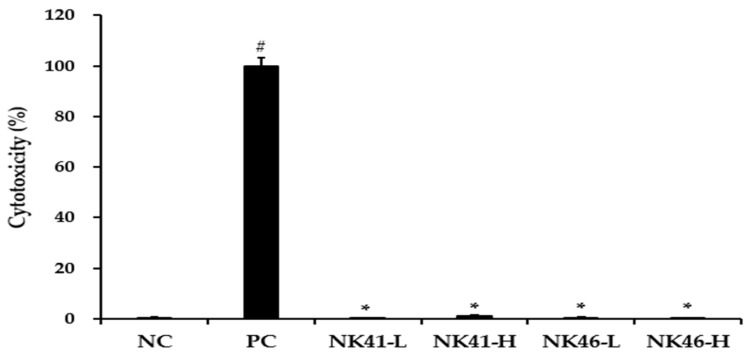
Cytotoxicity of *L. mucosae* NK41 and *B. longum* NK46 against Caco-2 cells. Data are presented as the mean ± standard deviation of three independent experiments (*n* = 3) and were analyzed using Student’s *t*-test. # *p* < 0.05 vs. NC and * *p* < 0.05 vs. PC. NC: negative control; PC: positive control; NK41-L: low dose (1 × 10^7^ CFU/mL) of NK41 treated cells; NK41-H: high dose (1 × 10^9^ CFU/mL) of NK41 treated cells; NK46-L: low dose (1 × 10^7^ CFU/mL) of NK46 treated cells; NK46-H: high dose (1 × 10^9^ CFU/mL) of NK46 treated cells.

**Figure 3:**
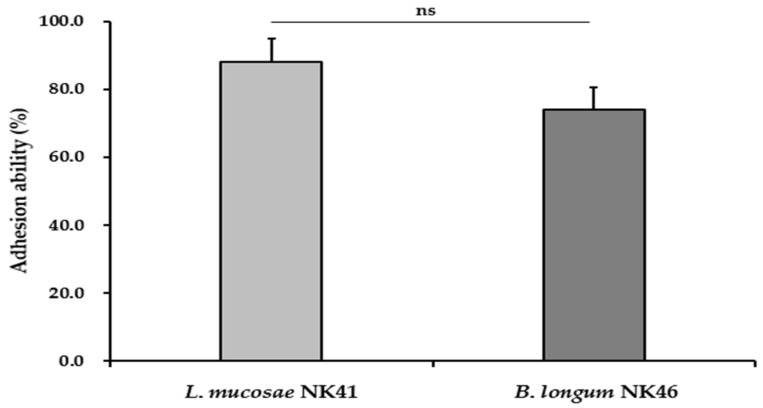
Adhesion ability of *L. mucosae* NK41 and *B. longum* NK46 to the intestinal Caco-2 cell line. Data are presented as the mean ± standard deviation of three independent experiments (*n* = 3) and were analyzed using Student’s *t*-test. ns: not significant.

The ability of probiotics to adhere to the host intestine is regarded as a pivotal factor in enhancing their health benefits [11]. Furthermore, their adhesion ability allows probiotics to prolong their survival in the GIT and promote interactions between the bacteria and the host [39]. Therefore, the ability of probiotics to adhere to intestinal epithelium is vital. To ensure the safety of tested probiotic strains, their cytotoxicity in Caco-2 cells was assessed prior to the adhesion ability test. *L. mucosae* NK41 and *B. longum* NK46 showed no cytotoxicity in Caco-2 cells at concentrations up to 1 × 10^9^ CFU/mL (Figure 2). Therefore, an adhesion ability test was conducted using a concentration of 1 × 10^8^ CFU/mL of the probiotic strains. As shown in Figure 3, the adhesion rates of *L. mucosae* NK41 and *B. longum* NK46 were 88.2 ± 6.7% and 74.1 ± 6.4%, respectively. No significant differences were observed between the strains in terms of the percentage of adhesion to Caco-2 cells. Consistent with these findings, previous studies have reported that commercial probiotics strains, including *Lactobacillus* spp. and *Bifidobacterium* spp., exhibit strong binding abilities to Caco-2 and HT-29 cells [40,41]. Furthermore, strains with robust adherence to Caco-2 cells effectively suppressed the adhesion of pathogens to Caco-2 cells [38]. These findings suggest that *L. mucosae* NK41 and *B. longum* NK46 may be capable of adhering to and colonizing the epithelium of the human GIT.

### 3.4. Profile of Enzyme Activities of the Probiotic Strains

Probiotics produce various beneficial enzymes that promote health. For example, glycoside hydrolase enhances the bioavailability of plant polysaccharide; β-galactosidase reduces lactose intolerance by breaking down lactose in intestine; and protease aids in digestion within the GIT by hydrolyzing proteins into peptides and amino acids [42]. However, probiotics should not produce β-glucuronidase, which is associated with cancer development through the reactivation of carcinogens [10].

A semi-quantitative assessment of enzymatic activity in the probiotic strains was conducted using the API ZYM kit, and the results are presented in Table 3. *L. mucosae* NK41 and *B. longum* NK46 exhibited the high activities of α-galactosidase and β-galactosidase. Although both strains showed positive results for α-glucosidase activity, β-glucosidase activity was only observed in *L. mucosae* NK41. The activities of alkaline phosphatase, lipase (C14), valine arylamidase, cystine arylamidase, trypsin, α-chymotrypsin, β-glucuronidase, N-acetyl-β-glucosaminidase, α-mannosidase, and α-fucosidase were not observed in either of the strains. In particular, because neither strain exhibited β-glucuronidase activity, it may be assumed that there are no safety concerns associated with the commercial application of either strain. The enzymatic profile of *L. mucosae* NK41 was similar to that of strains of *Limosilactobacillus reuteri* [10,43], the species most closely associated with *L. mucosae*, and that of *B. longum* NK46 resembled other *B. longum* strains [42].

### 3.5. Hemolytic and Gelatinase Activities of L. mucosae NK41 and B. longum NK46

**Figure 4:**
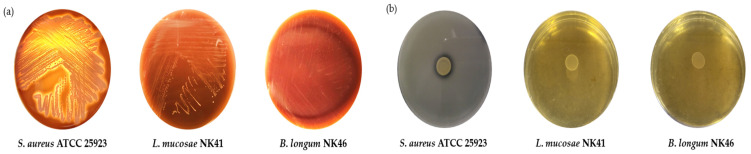
Hemolysis (**a**) and gelatinase activity (**b**) of *L. mucosae* NK41 and *B. longum* NK46. Staphylococcus aureus ATCC 25923, which is a pathogenic strain, was used as a positive control.

Considering hemolysis and gelatinase as virulence factors, the absence of these factors is a criterion for the selection of potential novel probiotic strains. Hemolytic activity induces the lysis of red blood cells and destruction of hemoglobin, resulting in anemia, fever, and skin rashes [44]. Pathogenic bacteria that produce gelatinase effectively attack the host by penetrating tissues through their gelatinase activity [17]. We evaluated the hemolytic and gelatinase activities of *L. mucosae* NK41 and *B. longum* NK46. Both strains exhibited γ-hemolysis, indicating no hemolytic activity, on 5% sheep blood agar plates (Figure 4a) and showed no clear zone on 3% gelatin agar plates (Figure 4b). Conversely, *S. aureus* ATCC 25923, which was used as a positive control, displayed β-hemolysis and gelatinase activity.

### 3.6. Antibiotic Susceptibility of the Probiotic Strains

Absence of antibiotic resistance is a key criterion for probiotic strains intended for food applications. Bacterial antibiotic resistance is achieved through intrinsic mechanisms, acquired mechanisms involving chromosomal mutations, or acquired mechanisms via horizontal gene transfer [45]. There is a high risk of transferring antibiotic resistance determinants from probiotic strains to pathogenic bacteria through plasmids, transposons, and other mobile genetic materials in cases of acquired resistance [23]. Therefore, probiotic strains should be sensitive to most antibiotics of human importance. In this study, the MICs of *L. mucosae* NK41 and *B. longum* NK46 were determined according to the CLSI guidelines, and the results were interpreted according to the EFSA guidelines. As shown in Table 4, both strains were sensitive to all the tested antibiotics. In previous studies, some strains of *L. mucosae* were found to be resistant to tetracycline, gentamicin, vancomycin, streptomycin, clindamycin, and erythromycin [11,23,29]. *B. longum* KABP042, isolated from the feces of healthy children, showed resistance to erythromycin and clindamycin [46]. Furthermore, Shin et al. [47] reported that many commercial probiotic strains exhibit resistance to various clinically relevant antibiotics. Nevertheless, according to a previous report, resistance of LAB to antibiotics, including *Lactobacillus* and *Bifidobacterium*, is typically natural and is not transmissible in most cases [48]. In this regard, because both strains used in this study were susceptible to two groups of antibiotics, including inhibitors of cell wall synthesis (ampicillin and vancomycin) and protein synthesis (gentamicin, kanamycin, streptomycin, erythromycin, clindamycin, tetracycline, and chloramphenicol), there was no concern regarding the potential dissemination of antibiotic resistance by both strains.

### 3.7. Lactate Production of the Probiotic Strains

Lactate exists in two stereoisomeric forms, namely the L-form and D-form. Some strains of LAB, including *Lactobacillus* and *Bifidobacterium* genera, produce both D(−)-lactate and L(+)-lactate [10,49]. Since D(−)-lactate cannot be metabolized in the human gut, the excessive production and accumulation by gut bacteria could potentially lead to D-lactate acidosis and short bowel syndrome [50]. In humans, the occurrence of D-lactate acidosis is rare, and has been reported exclusively in individuals with short bowel syndrome [10]. In this study, the ratio of D(−)-lactate to L(+)-lactate in *L. mucosae* NK41 and *B. longum* NK46 was determined using the enzymatic method, and the results of lactate production for these strains are shown in Table 5. *L. mucosae* NK41 produced 6.1 ± 0.2 mM (28.2 ± 0.7%) and 15.6 ± 1.1 mM (71.8 ± 0.7%) of D(−)-lactate and L(+)-lactate, respectively. Whereas *B. longum* NK46 produced 0.4 ± 0.1 mM (2.2 ± 0.4%) and 19.2 ± 0.5 mM (97.8 ± 0.4%) of D(−)-lactate and L(+)-lactate, respectively. These results are consistent with those of previous studies [51,52]. According to Lee et al. [51], some strains of *Lactobacilli* produced D(−)-lactate within the range of 31.4%–36.1%, which was lower than that of L(+)-lactate (63.9–68.6%). *B. longum* subsp. *infantis* CECT 7210 produced D(−)-lactate at a level of approximately 2.2% [52]. Furthermore, *B. lactis* BB-12^®^, certified as GRAS by the FDA, produced L(+)-lactate in excess of 95% [53]. The results of this study showed that both strains exhibited significantly higher levels of L(+)-lactate than D(−)-lactate, indicating that there are no safety concerns associated with their use as probiotics.

### 3.8. Detection of Virulence Factor Genes in the Probiotic Strains

**Figure 5:**
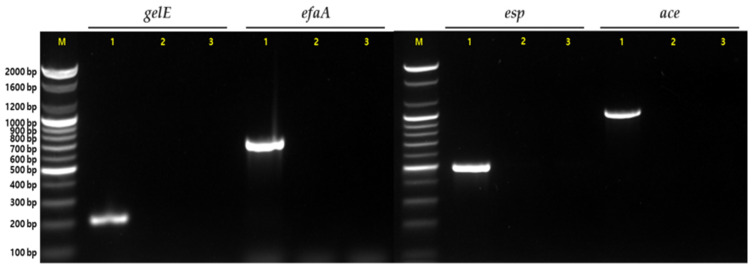
PCR detection of genes associated with virulence factors in *L. mucosae* NK41 and *B. longum* NK46. Lane M: molecular marker; lane 1: positive control; lane 2: *L. mucosae* NK41; lane 3: *B. longum* NK46.

Given the substantial impact of virulence factors on the risk of infection, assessing potential virulence genes in newly isolated probiotic strains is essential to determine their suitability for commercial applications as functional foods [11]. Therefore, we investigated whether the tested probiotic strains possessed virulence genes encoding *gelE*, *efaA*, *ace*, and *esp*, and the results are presented in Figure 5. The *gelE* gene, which is associated with gelatinase production [54], was not detected in *L. mucosae* NK41 or *B. longum* NK46, consistent with the in vitro findings of gelatinase activity. Furthermore, both strains were negative for *ace* and *efaA* genes. These genes are associated with the synthesis of different substances involved in microbial colonization and adhesion and may have detrimental effects on human infections [11]. Although these genes are commonly found in *Lactococcus* spp. and *Enterococcus* spp. [18], previous studies have shown that some *Lactobacillus* spp., including strains of *L. mucosae* and *L. plantarum*, harbor these genes [11,55]. Additionally, the *esp* gene for enterococcal surface proteins, which is associated with increased biofilm formation, virulence, and antibiotic resistance, was not detected in both strains [54,56]. Therefore, our study suggests that both strains have no safety concerns associated with their use as probiotics because they do not harbor the tested virulence genes.

## 4. Conclusions

Probiotics have attracted considerable interest in the functional food market due to the increased evidence of various beneficial effects in clinical trials. So, various users demand to use probiotic strains with efficacy and safety. In this study, we focused on the evaluation of probiotic properties and safety of *L. mucosae* NK41 and *B. longum* NK46 strains, isolated from human feces. Both strains exhibited resistance to simulated gastrointestinal environments and possess surface-binding properties, as well as the ability to adhere to intestinal epithelial cells, allowing colonization of the GIT. Furthermore, they may be considered safe due to their susceptibility to most common antibiotics and the absence of hemolytic activity or studied virulence genes. Therefore, these strains have the potential to be used as probiotics in food industry. Nevertheless, further studies may be conducted to validate their potential health benefits and applications.

## Figures and Tables

**Table 1:** Primer sequences used in the detection of genes encoding for virulence factors.

Target Gene	Virulence Factor	Primers (5′ to 3′)	Product Size (bp)	References
*gelE*	Gelatinase	TATGACAATGCTTTTTGGGAT AGATGCACCCGAAATAATATA	213	[11]
*ace*	Adhesion of collagen	GAATTGACAAAAGTTCAATCG GTCTGTCTTTTCACTTGTTTC	1008	
*efaA*	Endocarditis antigen	GCCAATTGGGACAGACCCTC CGCCTTCTGTTCCTTCTTTGGC	688	
*esp*	Enterococcal surface protein	AGATTTCATCTTTGATTCTTGG AATTGATTCTTTAGCATCTGG	510	

**Table 2:** Survival rate of *Limosilactobacillus mucosae* NK41 and *Bifidobacterium longum* NK46 under simulated gastrointestinal conditions.

Strains	Initial Counts (log CFU/mL)	SGF ^a^ (log CFU/mL)	SIF ^b^ (log CFU/mL)	Survival Rate (%)
*L. mucosae* NK41	7.65 ± 0.04	5.97 ± 0.03 *	5.46 ± 0.08 *	71.45 ± 0.76
*B. longum* NK46	7.84 ± 0.04	3.24 ± 0.11 *	3.20 ± 0.13 *	40.82 ± 1.41

Data are presented as the mean ± standard deviation of three independent experiments (*n* = 3). Asterisks (*) denote significant differences from the initial counts (*p* < 0.05), analyzed using Student’s *t*-test. ^a^ SGF: simulated gastric fluid; ^b^ SIF: stimulated intestinal fluid.

**Table 3:** Enzymatic activities of *Limosilactobacillus mucosae* NK41 and *Bifidobacterium longum* NK46.

Enzyme	*L. mucosae* NK41	*B. longum* NK46
Control (Negative)	0	0
Alkaline phosphatase	0	0
Esterase (C4)	3	2
Esterase Lipase (C8)	2	1
Lipase (C14)	0	0
Leucine arylamidase	3	4
Valine arylamidase	0	0
Cystine arylamidase	0	0
Trypsin	0	0
α-Chymotrypsin	0	0
Acid phosphatase	1	0
Naphtol-AS-BI-phosphohydrolase	2	2
α-Galactosidase	4	5
β-Galactosidase	5	5
β-Glucuronidase	0	0
α-Glucosidase	2	4
β-Glucosidase	5	0
N-acetyl-β-glucosaminidase	0	0
α-Mannosidase	0	0
α-Fucosidase	0	0

Data are presented on a scale of 0 (no reaction) to 5 (maximum activity).

**Table 4:** Antibiotic-resistant profiles of *Limosilactobacillus mucosae* NK41 and *Bifidobacterium longum* NK46.

Antibiotics ^a^	*L. mucosae* NK41			*B. longum* NK46		
	Cut-Off Value (μg/mL)	MIC ^b^ (μg/mL)	Susceptibility (S/R)	Cut-Off Value (μg/mL)	MIC (μg/mL)	Susceptibility (S/R)
AMP	2	0.5	S	2	0.125	S
VAN	n.r. ^c^	256	-	2	0.5	S
GEN	16	8	S	64	16	S
KAN	64	64	S	n.r.	64	-
STR	64	64	S	128	8	S
ERY	1	<0.125	S	1	<0.125	S
CLI	4	0.125	S	1	0.125	S
TET	8	4	S	8	2	S
CHL	4	4	S	4	0.5	S

Probiotic strains were categorized as susceptible (S) or resistant (R) based on the EFSA cut-off values. ^a^ AMP: ampicillin; VAN: vancomycin; GEN: gentamicin; KAN: kanamycin; STR: streptomycin; ERY: erythromycin; CLI: clindamycin; TET: tetracycline; CHL: chloramphenicol; ^b^ MIC: minimum inhibitory concentration; ^c^ n.r.: not required.

**Table 5:** D-/L-lactate production in *Limosilactobacillus mucosae* NK41 and *Bifidobacterium longum* NK46.

Strains	D-lactate (mM)	L-lactate (mM)	Ratio of Isomers (%)	
			D-Form	L-Form
*L. mucosae* NK41	6.13 ± 0.23	15.62 ± 1.08	28.20 ± 0.68	71.80 ± 0.68
*B. longum* NK46	0.43 ± 0.09	19.22 ± 0.50	2.20 ± 0.40	97.80 ± 0.40

Data are presented as the mean ± standard deviation of three independent experiments (*n* = 3).

## Data Availability

The data presented in this study are available on request from the corresponding author.
